# Literature Review of Current Management of Colorectal Liver Metastasis

**DOI:** 10.7759/cureus.3940

**Published:** 2019-01-23

**Authors:** Diana Mitchell, Yana Puckett, Quang N Nguyen

**Affiliations:** 1 Surgery, Texas Tech University Health Sciences Center, Lubbock, USA

**Keywords:** colorectal cancer, liver metastasis, management, surgery, chemotherapy, intra-arterial chemotherapy, radiofrequency ablation

## Abstract

Colorectal cancer is a leading cause of cancer mortality in the United States, and metastasis to the liver is a frequent sequela. Currently, surgical resection is the best option for curative treatment and/or long-term survival after colorectal liver metastasis (CRLM), but unfortunately, not all patients are surgical candidates. Alternative and adjunct therapies commonly used in the treatment of CRLM include chemotherapy, biologic therapy, radio-embolization, and radiofrequency ablation. The aim of this review was to report the various treatment modalities and outcomes currently used in the treatment of CRLM.

## Introduction and background

The World Health Organization (WHO) lists colorectal cancer (CRC) as the third leading cause of cancer and the fourth leading cause of cancer mortality in the United States [1]. In 2012, 14.1 million new CRC cases were diagnosed with 8.2 million deaths and 32.6 million living with the disease worldwide, and in the United States alone, approximately 130,000 new cases are diagnosed each year [1-2]. Due to the portal venous drainage from the colon, the liver is the most frequent site of metastases. Approximately 50% of patients are diagnosed with synchronous or metachronous colorectal liver metastases (CRLM), and it is the leading cause of death in CRC patients [2]. Liver metastases are present in 20% to 50% of patients upon initial diagnosis, and the remaining half of CRC patients will develop liver metastases throughout the course of their disease [2]. 

Currently, surgical resection is the best option for curative treatment or long-term survival after CRLM diagnosis [2-13]. Patients who only receive palliative therapy typically survive just seven to eight months. Survival in liver resected patients at five years is anywhere between 24% and 40%, with a median survival time of 28-46 months [2]. Unfortunately, not all patients are ideal candidates for surgical resection. This may be due to the number and location of metastases, instability of the patient, lack of sufficient unaffected liver, or comorbidities. In order to convert an unresectable case to a resectable one, many physicians utilize other treatment regimens in the hopes of reducing tumor size and giving the patient time to qualify as a surgical candidate.

When surgical resection is not deemed a viable option, locoregional therapies are increasingly practiced [14]. Regimens such as systemic chemotherapy, intra-arterial chemotherapy, and ablation are common adjuvant therapies utilized. While these treatment modalities are beneficial for many patients, they do not confer the same survival advantages as surgical resection when used alone. When a candidate is deemed unsuitable for surgery, the patient should be offered systemic chemotherapy and/or local ablative therapies as appropriate [2]. These therapies, when used in isolation or combination, are the current regimens utilized to treat CRLM. The aim of this article was to review the current literature on the management and treatment of CRLM.

## Review

Patient assessment

When initially diagnosed with CRLM, the patient should be evaluated by a multidisciplinary team including medical oncologists and diagnostic and interventional radiologists, as care for this patient population is complex [2,12,14]. Suspicion of metastatic disease should always be assessed with radiological imaging such as computed tomography (CT) scan, magnetic resonance imaging (MRI), or ultrasonography followed by subsequent histological confirmation when appropriate [2,11-12]. High-quality contrast-enhanced imaging should determine the location of the hepatic lesions and their relationship to the main hepatic vessels and the biliary tree [2,11]. Liver function should be assessed with complete blood examination including alanine aminotransferase (ALT), aspartate transaminase (AST), total bilirubin, prothrombin time, and albumin levels [2,15]. The patient should be assessed for the presence of ascites, cirrhosis, hepatitis, or any other liver abnormalities. Overall health status, organ function, and concomitant non-malignant disease must be evaluated [12]. Any medical comorbidities should also be considered.

Whenever feasible, surgical resection remains the treatment of choice for isolated CRC liver metastases [2-12]. If surgical resection seems feasible, the volume of the future liver remnant (FLR) should be calculated to avoid postoperative liver insufficiency. Although there is no absolute consensus regarding the minimum acceptable FLR, resection should be recommended only if sufficient liver parenchyma to maintain liver function is expected [12,14]. Depending on the quality of the liver, the minimum volume of the FLR varies. Guidelines suggest that in a healthy liver, the FLR should be at least 20% of total liver volume, with some degree of liver dysfunction at least 30%, and with cirrhosis 40% or more depending on the degree of dysfunction [2,14]. If FLR is insufficient, portal vein embolization (PVE) of the segments planned for resection can induce hepatocyte growth on the contralateral side and increase FLR [2,12]. Repeat volumetry should be repeated in four weeks to reassess FLR [2,15]. If FLR is acceptable, and the patient meets safety criteria, surgical resection should be planned. Additional strategies to achieve a resectable state include downstaging or conversion chemotherapy, two-stage resection, and associating liver partition with portal vein ligation for staged hepatectomy [12]. When surgical resection is not feasible, alternative strategies may be considered, such as systemic chemotherapy, ablation, and transarterial embolization.

Surgical resection

Operative approaches include open technique, laparoscopy, and anatomic and nonanatomic resection. Surgical resection has been shown to reduce mortality and morbidity from CRLM and is the preferred treatment whenever possible [2-13]. Liver resection is the most effective treatment to achieve long-term survival and offers the possibility of a cure for CRC patients with liver metastases [12]. When compared to isolated liver perfusion therapy, surgical resection results in higher and/or equal survival rates, even when complications occur after resection [16].

The goal of this treatment modality should be to remove all the metastases with microscopically negative margins, although the width of the negative surgical margins has not been shown to be associated with increased risk of local recurrence. Recently, it has been determined that the width of the resection margin does not impact survival as long as it is negative [14]. In all cases of hepatectomies, sparing of the parenchyma is preferred as patients have decreased morbidity and higher rates of salvageability in cases of recurrence [2].

In a retrospective review of 1,600 patients who underwent hepatic resection for metastatic CRC, patients were analyzed from 1985 to 2004. Patients were put into two eras: era I being 1985 to 1998 and era II being 1999 to 2004. Median disease-specific survival (DSS) increased from 43 months in era I to 64 months in era II, and five-year DSS increased from 37% in era I to 51% in era II [7]. Hepatic resection of patients with CRLM has also been shown to increase long-time survival, and in cases with one CRLM, has increased survival to similar levels as CRC patients who do not develop CRLM [6,17]. A 10-year population-based analysis of 5,772 cases of primary colorectal adenocarcinoma showed significant survival benefits for CRLM resection upon univariate and multivariate analysis when adjusted for age, sex, year of resection, time of CRLM diagnosis, and number of CRLM [6]. In a retrospective review, from 1990 to 2006, the five-year overall survival (OS) increased from 9.1% in the earliest time period (1990-1997) to 19.2% in the 2001-2003 time period, and improved outcomes from 1998 to 2004 were found to be a result of an increase in hepatic resection performed in 20% of patients [17]. Overall, in patients with CRLM, approximately 19% to 40% of patients remain alive five years after resection with a median survival time of 28 to 46 months, and around two-thirds of surviving patients are disease free, compared to those who only undergo palliative treatment surviving seven to eight months [2,5,10].

Synchronous CLM

Synchronous diagnosis of CRC and CRLM can bring into question the timing and sequence of surgical intervention. Simultaneous resection is generally acceptable when the colon surgery is straightforward and there are only minor liver metastases [2]. In cases of complex colorectal procedures with the need for major liver resection, it is generally advised to address one anatomical region at a time [2]. A systematic review of the surgical management of synchronous CRLM found that simultaneous resection of CRC and CRLM, CRC resection followed by CRLM resection, and CRLM resection followed by CRC were equally acceptable management strategies and that no strategy appeared inferior to the others. Studies included in the review did favor simultaneous approach on the basis of length of hospital stay. Duration of procedure, blood loss, morbidity, and perioperative mortality varied among the studies included in the review [8]. In cases where simultaneous resection is not advisable, resection of the liver first may be an attractive option, as the liver is usually the determining factor for complete disease resection. In other cases where the primary tumor is symptomatic, it may need to be addressed first, followed by hepatic resection [2]. The choice of which approach to take will also depend on the degree of liver metastases, symptoms of the patient, the possibility of the primary CRC or CRLM progressing to an unresectable stage, and adjuvant therapies being used.

Two-stage hepatectomy

A two-stage hepatectomy may be advised when the patient presents with an initially unresectable bilobar CRLM that is amenable to metastasectomy not requiring hilar dissection. An initial course of chemotherapy is administered, typically four to six cycles. This is followed by repeat imaging, and if there is a response, or the disease is stable, a stage one resection is completed of the future FLR. There may be a need to complete a PVE for hypertrophy of the FLR after this stage, and it is, therefore, important to have resected all disease in the future FLR to avoid tumor growth following PVE. After four to six weeks, with or without chemotherapy treatment, liver regeneration is assessed through repeat imaging. If satisfactory, stage two resection is completed to remove the remaining metastases. As first stage resection has no survival benefit, it is important to minimize morbidity after the first stage to allow patients to complete the second stage. Three-year OS ranges from 50% to 84% for patients who complete both stages of resection [18].

Extrahepatic disease

In patients with extrahepatic disease (EHD), hepatic resection may be a viable treatment modality. This is only so in cases where the EHD is limited and resectable [2]. In a retrospective review, the survival of 840 patients resected for CRLM, who also had resectable EHD, was compared with the survival of patients without EHD. Patients resected for CLM with concomitant EHD experienced a statistically significant lower five-year survival than those without EHD (28% vs 55%). Multivariate analysis showed five poor prognostic factors: EHD location other than lung metastasis, EHD concomitant to CRLM recurrence, carcinoembryonic antigen level at least 10 ng/mL, at least six CLM, and right colon cancer. In the EHD group, patients with an EHD recurrence experienced better outcomes when resected than those treated by chemotherapy alone [19].

A systematic review of 22 studies found that surgical resection of CRLM and concomitant EHD in carefully selected patients may achieve survival results superior to non-surgically treated patients. Patients with lung metastases had the longest median survival (41 months), followed by portocaval lymph node and peritoneal metastases (both 25 months). The median disease-free survival was 12 months, median OS was 30 months, the median five-year survival rate was 19%, and the median five-year survival of patients with R0 hepatectomy with resection of EHD was 25% [5]. Although EHD is considered a contraindication to surgery in many cases, in highly select patients where complete resection of CRLM with EHD is possible, survival rates may be superior to non-surgically treated patients.

Overall, surgical resection of CRC metastasis remains the treatment of choice. Severe postoperative complications include bile leak and perihepatic abscess, and patients should be monitored closely after surgery. Routine liver resection is not recommended in patients with a portal nodal disease or those with non-pulmonary extrahepatic metastases which cannot be completely resected [12]. In selected patients with large tumors, surgical resection may be combined with adjuvant therapies, such as ablation, in order to increase the chances of long-term survival without reoccurrence [2]. 

Chemotherapy

Chemotherapy is commonly used as adjuvant therapy in the treatment of CRLM. When surgical resection is not feasible, chemotherapy may be used as primary treatment, or as a bridge to surgical intervention. Conventional agents include fluorouracil (5-FU), leucovorin, capecitabine, oxaliplatin, and irinotecan [14]. Fluorouracil-based chemotherapy is the foundational treatment and is usually administered in combination with other agents. Examples include FOLFOX (folinic acid (FA), 5-FU, and oxaliplatin) and FOLFIRI (FA, 5-FU, and irinotecan) [20]. These agents are used to disrupt the cell cycle through various mechanisms and result in cell death of rapidly dividing malignant cells.

Chemotherapy following liver resection aims to reduce recurrence of CRLM. Additionally, chemotherapy may be used postoperatively to address micrometastatic disease that is too small to be seen on imaging, but it has not been shown to improve survival when used in this manner [2]. 

However, caution must be used as chemotherapy may result in significant injury to the liver and lead to postoperative complications. As such, it should be limited in duration whenever possible. Individualized treatment, such as periods of aggressive chemotherapy interspersed with maintenance periods, should be tailored to patient response and needs as appropriate [14].

Chemotherapy and surgery

Perioperative chemotherapy, either before and after resection, or after resection only, is recommended in patients with resectable liver metastatic disease [12]. When combined with surgery, chemotherapy may be an effective treatment modality, and the use of systemic chemotherapy in combination with surgical resection has become an accepted standard of care for patients with CRLM [20]. The current recommendation is to perform metastasectomy two to three months following preoperative chemotherapy or when the metastases become resectable [21].

Although a standard of care, studies have shown that chemotherapy combined with surgery may not improve OS. In a systematic review of systemic adjuvant, neoadjuvant, and perioperative chemotherapy for resectable CLM, the authors found no significant improvement in median OS from chemotherapy and surgery when compared with surgery alone [20].

In a randomized, controlled, phase three trial, 364 patients were randomly assigned to either perioperative chemotherapy with FOLFOX or surgery only. In the perioperative chemotherapy group, median OS was 61.3 months and five-year OS was 51.2%. In the surgery-only group, median OS was 54.3 months and five-year OS was 47.8%. Despite these differences, there was no statistical difference in OS with the addition of perioperative chemotherapy with FOLFOX compared with surgery alone in patients with resectable CRLM. However, FOLFOX in combination with liver resection did appear to increase disease-free survival (DFS) when compared to surgery alone. Despite the lack of difference in OS between the groups, the authors argued for the continued use of perioperative chemotherapy as previous studies have shown an observed benefit in progression-free survival [7].

On the other hand, a retrospective study of adjuvant chemotherapy post-resection of CRLM showed that adjuvant chemotherapy prolonged relapse-free survival (RFS) and OS in patients who received preoperative chemotherapy. In this study, 163 patients received preoperative chemotherapy followed by metastasectomy, and 100 of those patients received adjuvant chemotherapy with the remaining 63 patients receiving no adjuvant chemotherapy. After adjusting for risk factors (metachronous/synchronous metastases, differentiated grade of the primary tumor, number of metastases, size of the largest metastasis, duration of preoperative chemotherapy, radiologic response, and pathologic response), the adjuvant chemotherapy group was estimated to have a 54% RFS and a 55% OS advantage compared to patients without adjuvant chemotherapy [21]. Thus, patients who have received preoperative chemotherapy should still be considered for adjuvant chemotherapy.

In an observational study, 53 patients with $\geq$ three CRLM received preoperative chemotherapy followed by resection, while 96 patients with $\geq$ three CRLM underwent resection first followed by chemotherapy. Patients who received preoperative chemotherapy had a three-year DFS rate of 31.7% compared to 20.4% in the postoperative chemotherapy group. Upon various multivariate analyses, the preoperative chemotherapy group continued to have better DFS rates, and the authors recommended preoperative chemotherapy be preferentially considered for patients who experience difficulty undergoing complete resection for multiple CRLM [22].

Bridge to surgery

The majority of CRLM patients have metastatic disease that initially is not suitable for resection [9]. In these cases, chemotherapy can be a useful tool in the attempt to convert an unresectable case to a resectable one. Chemotherapy has been shown to reduce the bulk of metastatic disease, and patients with initially unresectable CRLM who have a sufficient downstaging response to conversion chemotherapy should be recommended for liver resection as soon as possible [12,14].

In a study of 114 patients with non-resectable CRLM, patients were randomly assigned to receive FOLFOX or FLOFIRI. After a retrospective review, resectability rates increased from 32% at baseline to 60% after chemotherapy [23]. In another study, CRLM downstaging was shown to allow up to 12.5% of unresectable cases to undergo secondary hepatic resection after an average of ten courses of chemotherapy [3]. Following chemotherapy, if the patient has stable disease with an adequate liver remnant, then resection is the treatment of choice [23]. Current guidelines also recommend an adjuvant therapy after CRLM resection, and systematic chemotherapy with 5-FU±oxaliplatin has been shown to confer a survival advantage [4].

Systemic therapy

Recent advances in systemic chemotherapy have led to high response rates, but unfortunately complete clinical responses are rare when used in isolation and five-year survival rates are typically less than 1% [2,12]. First-line palliative chemotherapy is a fluoropyrimidine in various combinations and schedules. Combination therapy with FOLFOX or FLOFIRI has been shown to provide higher response rates than 5-FU/leucovorin, but combination therapy was not found to be superior to sequential treatment in terms of OS. Frail patients, who may not tolerate combination therapy, may be adequately treated with monotherapy. Second-line chemotherapy includes monoclonal antibodies against vascular endothelial growth factor (VEGF) and against epidermal growth factor receptor (EGFR) in combination with chemotherapy. This combination therapy has been shown to improve the outcome of metastatic CRC patients. The optimal duration of chemotherapy has not been determined but is currently recommended for a fixed period of three to six months or until progression or toxicity [4,11]. Despite advances in systemic chemotherapies, long-lasting comprehensive clinical responses are rare when treating with chemotherapy alone [2].

Chemotherapy is also associated with hepatotoxicity. Complications such as steatosis, steatohepatitis, and sinusoidal distention increase the risk of liver resection [2]. A systematic review study utilizing preoperative chemotherapy for CRLM showed hepatic steatosis after treatment with 5-FU, non-alcoholic steatohepatitis after treatment with irinotecan, and hepatic sinusoidal obstruction syndrome after treatment with oxaliplatin. These hepatic changes can affect patient outcome and can increase morbidity and mortality following treatment with chemotherapy and/or CLM resection [24]. When selecting a chemotherapeutic agent, both toxicity and expected response rate should be considered.

Chemotherapy-biologic therapy

The desire to convert non-resectable cases to resectable has driven the development of modern cytotoxic chemotherapy used in combination with monoclonal antibodies against growth factors and growth factor receptors. Examples include cetuximab, a monoclonal antibody against EGFR and bevacizumab, a humanized recombinant monoclonal antibody that blocks the activity of VEGF.

In a randomized, open-label, multicenter phase III trial, cetuximab was combined with FOLFIRI and used as first-line therapy for CRLM. The study found that first-line treatment with cetuximab combined with FOLFIRI, compared with FOLFIRI alone, reduced the risk of progression of metastatic CRC, but was limited to patients with KRAS wild-type tumors. Adverse reactions were more frequently in the cetuximab group and included sink reactions, infusion-related reactions, and diarrhea [25].

In a randomized two-by-two factorial design, bevacizumab was added to first-line capecitabine plus oxaliplatin (XELOX) or FOLFOX in patients with metastatic CRC. Median PFS was significantly improved in the bevacizumab group (9.4 months) when compared to the placebo group (eight months), but median OS was not statistically significant (21.3 months in the bevacizumab group and 19.9 months in the placebo group). The response rate was not improved by the addition of bevacizumab. A high proportion of patients discontinued study treatment because of adverse events in the bevacizumab-containing arms compared to the placebo-containing arms (30% vs 21%). Adverse events of special interest to bevacizumab included thromboembolic events, hypertension, bleeding, gastrointestinal perforations, wound-healing complications, fistula/intra-abdominal abscess, and proteinuria [26].

Chemotherapy-biologic therapy is associated with hepatic and systemic toxicity and may increase morbidity and mortality post resection of CRLM, but careful monitoring of cumulative dose and liver blood tests will aid in keeping toxicity and morbidity to a minimum [27].

Intra-arterial chemotherapy

Early-stage tumor lesions, such as CRLM, are primarily supplied by the hepatic arteries [28]. Chemotherapy may be administered through the hepatic artery instead of systemically. This route confers the advantage of producing high concentrations of the therapeutic agent in the liver with minimal systemic toxicity. Hepatic artery infusion (HAI) of antineoplastic drugs has demonstrated favorable pharmacokinetic characteristics with high extraction ratios and high local drug concentrations. Additionally, systemic levels of antineoplastic agents following HAI are less than those following peripheral venous infusion (PVI). HAI with floxuridine shows 94% to 99% of the drug is extracted in the liver, with systemic levels approximately 25% of those following PVI, and 5-FU HAI shows systemic levels ranging from 50% to 77% of those following PVI [28].

HAI chemotherapy has been shown to convert the unresectable CRLM cases to the resectable ones and improve disease-free survival in patients who fail first-line chemotherapy [29]. It is used as palliative treatment in patients with unresectable cases, adjuvant therapy before resection, and as a bridge to liver transplantation [2].

In one study comparing HAI chemotherapy with systemic chemotherapy, patients treated with HAI had significantly longer OS. Median survival was 22 months compared to 15 months, and there was a statistically significant difference in progression-free survival with seven months in the HAI group and four months in the systemic group [30].

When combined with systemic chemotherapy, HAI has increased tumor response rates up to 80% when used as a first-line treatment and 50% as a second-line treatment. HAI has also been shown to decrease the risk of relapse of CRLM following resection by increasing DFS when combined with systemic chemotherapy [28].

Port-catheters are surgically implanted and connected to a subcutaneous port to enable easy access and multiple administrations of chemotherapeutic agents. Catheter implantation requires an experienced team, and as such may limit the prevalence of HAI therapy for CRLM patients [28]. Complications after several months of treatment include arterial obstruction, catheter thrombosis, catheter migration, and broken catheter with an overall complication rate of 29% [31].

Transarterial chemoembolization

Transarterial chemoembolization for liver metastases (TACE) is similar to HAI but also includes agents used to embolize small branches of the hepatic artery. TACE can be completed using various agents such as oil emulsion, beads, or microspheres [2]. When using irinotecan drug-eluting beads, 30 days after TACE, carcinoembryonic antigen (CEA) was reduced by 50% in one study and another found an 80% response rate with reduction of lesional contrast enhancement in all responding patients [32-33]. Irinotecan drug-eluting beads have also been shown to be an effective palliative therapy for unresectable and chemotherapy-resistant CRLM with median survival ranging from 13.3 to 25 months [34-35].

Radio-embolization

Selective internal radiation therapy (SIR-spheres) is the injection of microspheres carrying a dose of yttrium-90 (90Y) [2]. Radio-embolization is used to deliver localized radiation therapy to inoperable primary and secondary hepatic malignancies, such as CRLM. It may be used simultaneously with other therapies to improve response rates or as monotherapy [36].

Following treatment with radio-embolization, OS has been shown to be 12 months, median duration of response 8.3 months, and median time to progression 5.3 months [37-38]. In a prospective, multicenter, phase II clinical trial, patients with advanced, unresectable and chemorefractory CRLM were treated with SIR-spheres with a median OS of 12.6 months and a two-year survival of 19.6%. Thus, patients with liver-only, or liver-dominant CRLM who are chemotherapy refractory and who remain fit should be considered for salvage therapy using radio-embolization [36].

Adverse effects of radio-embolization are due to the small size of the microspheres and their ability to enter systemic circulation [2]. In one study, most adverse effects were classified as mild or moderate with one death due to acute renal failure and another due to liver failure. Both deaths were classified as possibly related to treatment [36].

Contraindications to intra-arterial chemotherapy include advanced liver disease, active GI bleeding, biliary obstruction, encephalopathy, refractory ascites, vascular invasion or portal vein invasion from the tumor, extrahepatic metastases, portosystemic shunts, and contraindication to arterial intervention. Complications include post-embolization syndrome, hepatic insufficiency or failure, and cerebral or pulmonary embolism/infarction [2].

Radiofrequency ablation

Another currently accepted adjuvant therapy for CRLM is liver-directed locoregional ablative therapy that may be conducted percutaneously or through operation. An ablation electrode is placed in the tumor, or tumor bed post resection, with the goal of creating a zone of coagulative necrosis that includes the tumor cells and adjacent parenchyma [2]. The most commonly used technique is radiofrequency ablation (RFA). This is commonly utilized in patients who do not meet resection criteria as it can increase the chance for survival and control local spread. RFA may also be used in combination with surgery and chemotherapy.

Patients with CRC metastases restricted to the liver are the best candidates for RFA, and tumors should be less than 5 cm, as larger tumors are associated with a high risk of recurrence, likely due to incomplete ablation [2]. Caution should be used when placing electrodes, as injury to adjacent structures could lead to sequelae such as central bile duct damage and heart arrhythmias.

In a study evaluating recurrence and outcomes in patients treated with CRLM resection only, RFA only, and RFA plus resection, liver-only recurrence after RFA was found to be four times the rate of resection only. It also showed that RFA alone or in combination with resection provided survival only slightly higher than nonsurgical treatment and that hepatic resection provided the highest survival rates [39]. In a retrospective study comparing CRLM resection, RFA, and combination resection with RFA, the treatment modality was statistically significant for OS on univariate analysis. The resection-only group had a median survival of 3.8 years and five-year OS of 43%, the RFA group had a median survival of 2.6 years and five-year OS of 23%, and the combination group had a median survival of 2.3 years. Median time to recurrence was 11 months for the resection group, seven months for the RFA group, and eight months for the combination group [40]. Consequently, RFA cannot be considered an equivalent procedure to hepatic resection, and if a CRLM is resectable, RFA may or may not be beneficial [39, 40]. In contrast, a systematic review of RFA showed that RFA prolonged time without toxicity and survival when used as an adjuvant therapy to surgical resection in well-selected patients [41].

In unresectable cases, RFA can be a safe and effective method for treatment [42-43]. In a prospective evaluation of 235 patients with CRLM who were not candidates for resection and/or failed chemotherapy, RFA yielded a three-year survival of 20.2% and five-year survival of 18.4% [44]. In a systematic review, median OS after RFA ranged between 24 and 59 months [41]. In another study, 262 patients with CRLM were treated with RFA with a median survival of 41 months in patients with metastatic lesions ≤3 cm and 21.7 months for those with lesions >3 cm. In this study, small lesion size proved to be the most favorable prognostic factor for survival [43].

Ablation with systemic chemotherapy can also be an effective treatment for unresectable CRLM. Because out-of-field recurrences are common with RFA, systemic or regional chemotherapy could be of use [42]. When comparing systemic chemotherapy alone to systemic chemotherapy plus RFA, promising results have been noted. In a randomized phase II trial, OS was compared between two treatment groups. The systemic group was treated with systemic chemotherapy and the combined group was treated with systemic treatment plus RFA±resection. Median OS was 45.6 months in the combined group and 40.5 months in the systemic group showing that aggressive treatment can prolong OS [45].

To date, there is only one randomized study on the efficacy of RFA. The European Organization for Research and Treatment of Cancer randomly assigned 119 patients with non-resectable colorectal liver metastases into two groups. One with systemic chemotherapy treatment and the other systemic treatment plus RFA. Progression free survival (PFS) at three years was 27.6% for the combined RFA plus systemic treatment group and 10.6% for the systemic only treatment group. Median PFS was 16.8 months for the combined group and 9.9 months for the systemic group, showing RFA plus systemic treatment results in significant progression-free survival of patients with unresectable CRLM. Due to trial downsizing, the ultimate effect of RFA on OS remains uncertain [46].

Stereotactic body radiation therapy

Stereotactic body radiation therapy (SBRT) is another technique utilized in the treatment of CRLM, especially for patients who are not surgical candidates. Before SBRT, radiation therapy was limited in the treatment of liver metastases due to the liver’s high sensitivity to radiation. When normal liver tissue is exposed to radiation, there is a risk of radiation-induced liver disease (RILD), which can lead to liver failure and death [47]. Conventional radiation applies low dose fractions to a large tissue volume, whereas SBRT delivers high-dose radiation directly to the tumor and maximizes normal-tissue sparing. During SBRT, a single high dose of radiation is applied precisely to the tumor, and only a few fractionated radiation treatments are required.

Planning for SBRT involves diagnostic imaging to locate areas of metastasis. SBRT is indicated for patients unable to tolerate surgery or if the tumor is difficult to remove. Patient selection is multifactorial and includes factors such as the number of lesions, lesion diameter, distance from other organs, liver function, and the free liver volume [48].

Studies have shown that SBRT is a well-tolerated and effective treatment for patients with CRLM. A study published in 2018 evaluated the clinical outcomes of 427 patients with 568 liver metastases from 25 academic and community-based centers. At a median follow up of 14 months, the median OS was 22 months and median OS was highest in patients with colorectal carcinoma (27 months). This study concluded that SBRT provided good overall survival as well as local control for metastatic liver lesions [49]. Additionally, a 2014 literature review analyzed the role of SBRT in treating liver metastases and concluded that SBRT is efficacious in treating patients with liver metastases who are not surgical candidates [50].

SaveSave

SaveSave

SaveSave

SaveSave

## Conclusions

Surgical resection of colorectal metastases is associated with improved survival and reduced mortality and morbidity. As such, it is the treatment of choice for CRLM. The majority of CRLM patients have initially unresectable cases, therapies utilized to downstage unresectable cases are essential. Chemotherapy may be used as primary treatment in unresectable cases, as a bridge to surgery in unstable patients, or a method to convert unresectable cases to resectable ones.

Systemic chemotherapy used in isolation has rare clinical response rates and thus should be used in combination with other treatment modalities, primarily surgical resection when possible. HAI of chemotherapeutic agents has been shown to increase OS, convert unresectable cases to resectable, and decrease systemic levels, thus reducing the side effects of chemotherapy. Although the addition of biologic agents to chemotherapy has shown promise, adverse effects may negate the benefits, and patients should be carefully monitored for toxicity and morbidity when utilizing this treatment modality.

Radio-embolization can be used as an alternative therapy when CRLM is treatment refractory, or simultaneously with other therapies, and has been shown to increase OS. RFA can increase survival and decrease the local spread of CRLM in unresectable cases. RFA alone is associated with higher recurrence rates, but when combined with resection it has been shown to increase OS. 

Studies have shown that SBRT is an efficacious mode of treatment for patients who are not surgical candidates. Unlike traditional radiation therapy that applies low fractions of radiation to a large tissue volume, SBRT delivers high-dose radiation precisely to the tumor. 

Management and treatment of CRLM is a complicated process and current literature advocates for surgical resection as the primary intervention. Further research is needed to explore other treatment modalities in the hopes of finding methods to increase OS, DFS, and PFS to rates similar to those of surgical resection. Providers treating patients with CRLM should be aware of current treatment options and utilize the various interventions according to their patients’ individual cases.
